# Transcriptome Analysis Reveals the Molecular Mechanisms for Mycorrhiza-Enhanced Drought Tolerance in Maize by Regulating the Ca^2+^ Signaling Pathway

**DOI:** 10.3390/jof11050375

**Published:** 2025-05-14

**Authors:** Qiaoming Zhang, Wenjing Yang, Miaomiao Wang, Junwei Chen, Zhaoran Zhang, Yanan Wei, Qingshan Chang, Minggui Gong

**Affiliations:** 1 College of Horticulture and Plant Protection, Henan University of Science and Technology, Luoyang 471023, China; zhangqm1013@163.com (Q.Z.);; 2 College of Food and Bioengineering, Henan University of Science and Technology, Luoyang 471023, China

**Keywords:** arbuscular mycorrhizal fungi, drought stress, differentially expressed genes, maize, RNA-seq

## Abstract

With the continuous change of climate, drought stress has emerged as the primary constraint on crop growth, posing a significant threat to the stability of global grain reserves. Arbuscular mycorrhizal fungi (AMF), as a kind of widely distributed root endophytes, enhance the drought tolerance of maize (*Zea mays* L.) through regulating the physiological and molecular responses. However, comprehensive transcriptome analysis to reveal the molecular mechanism of drought tolerance in the symbiotic process between AMF and maize is still limited. In the potted plant experiment, maizes inoculated with and without arbuscular mycorrhizal fungus *Funneliformis mosseae* were grown under well-watered (WW) or drought-stressed (DS) conditions. By using RNA-Seq and transcriptome analysis on maize roots and leaves, this work aimed to investigate the differential expressed genes (DEGs) related to the Ca^2+^ signaling pathway induced by AMF symbiosis under drought stress. Our findings indicated that *F. mosseae* inoculation resulted in a decrease in the net fluxes of Ca^2+^, while simultaneously elevating Ca^2+^ contents in the maize roots and leaves under well-watered or drought-stressed conditions. Notably, 189 DEGs were regulated not only by AMF symbiosis and drought stress, but also exhibited preferential expression in either leaves or roots. The annotation and enrichment of Gene Ontology (GO) and Kyoto Encyclopedia of Genes and Genomes (KEGG) showed that most of the DEGs were significantly enriched in Ca^2+^ signaling pathway genes, related to signal transduction, cellular process, and defense response. A high number of DEGs with this function (including calcineurin B-like protein (CBL), CBL-interacting protein kinase (CIPK), mitogen-activated protein kinase (MAPK), and calcium-dependent protein kinase (CDPK) receptor kinases) were upregulated-DEGs or downregulated-DEGs in *F. mosseae*-inoculated maizes under drought stress. Furthermore, some DEGs belong to transcription factor (TF) families, including bHLH ERF, and, MYB, were speculated to play key roles in improving the drought tolerance of maize. Based on the expression data and co-expression analysis between TF and Ca^2+^ signaling pathway genes, *Whirly1* with *CBL11*, and BRI1-EMS-SUPPRESSOR 1 (*BES1*) with *CBL10*, *CIPK24*, *CDPK1*, *CDPK14*, *CDPK19*, and *MAPK9* genes showed significant positive correlations, while *B3* domain-containing transcription factors (*B3* TFs) with *MAPK1* and both *CBL9* genes showed significant negative correlations in response to both *F. mosseae* inoculation and drought stress. The regulation of Ca^2+^ signaling pathways by AMF symbiosis was an important response mechanism of maize to improve their drought resistance. This study provides insightful perspectives on how AMF-induced modulation of gene expression within the Ca^2+^ signaling pathway can enhance the drought tolerance of mycorrhizal maize in the future.

## 1. Introduction

With global climate change and the expansion of human activities, the elevation of temperatures and decreased rainfall are becoming more severe and frequent in agricultural production areas, which exacerbates global agricultural drought events [1]. Drought stress is recognized as one of the most devastating abiotic factors limiting crop growth, development, and yield in arid and semi-arid regions, ultimately posing a significant threat to the stability of global grain reserves [2]. To alleviate the negative effects of drought deficiency, crops are needed to respond quickly by altering their physiological and biochemical processes, including photosynthesis, nutrient absorption, hormone balances, and cellular signal transduction [3,4]. Drought stress induces the Ca^2+^ signal pathway to stimulate a wide variety of cellular responses by mediating the intracellular Ca^2+^ concentration of different cell types, Ca^2+^ sensors, and their interacting proteins [5,6]. When exposed to drought stress, crops utilize calcium (Ca^2+^) as a secondary messenger to activate downstream biological responses and to regulate various signal transduction pathways, which rely on the transcriptional alterations of Ca^2+^ signaling genes and kinase activity of Ca^2+^ sensors to orchestrate their response [7]. These Ca^2+^ signatures are decoded by Ca-dependent kinases such as calcineurin B-like proteins (CBLs), CBL interacting protein kinases (CIPKs), mitogen-activated protein kinases (MAPKs), calcium-dependent protein kinases (CDPKs), calmodulins (CaMs), and calmodulin-like proteins (CMLs), which play key roles in crop growth and reproduction during abiotic and biotic stresses, as well as in the response to microbial signals during symbiotic interactions [8,9]. For example, *Zea mays ZmCIPK8* interacting with *ZmCBL1*, *4*, and *9*, and *ZmCIPK8* overexpression were proven to improve the drought tolerance of maize [10]. *Triticum aestivum TaCBL4*-*TaCIPK5* improved wheat resistance to *Puccinia striiformisf*. sp. *tritici* by activating the reactive oxygen species (ROS)-dependent system [11].

Maize (*Zea mays* L.) is one of the widely planted food and feed cereal crops throughout the world, and the total area of maize cultivation worldwide reaches about 160 million hectares in 2022, with its output accounting for 25% of the world’s total grain production [2]. According to FAOSTAT, the annual global production of maize exceeds one trillion tons, with projections indicating a potential doubling of demand by 2050 [12]. This crop has the advantages of rapid growth and strong adaptability, yet it is particularly susceptible to water deficit, and demands higher water intake during its initial establishment, vegetative and reproductive stages [13]. In China, the majority of maize cultivation areas lack irrigation and are almost entirely dependent on rainfall, and drought is the most significant limiting factor for maize production in numerous regions [2]. Drought stress poses a serious threat to seed germination and seedling growth, inducing premature flowering and prolonging the anthesis-silk interval, ultimately leading to substantial reductions in maize yield on a large scale [12]. Consequently, it is often beneficial to apply targeted agricultural techniques to enhance the drought resistance of maize in geographically drought-prone regions. Among these strategies, the utilization of arbuscular mycorrhizal fungi (AMF) stands out as a promising approach, which is an efficient and environmentally friendly measure for improving the plant resistance to drought stress [3].

AMF, as a prevalent group of root endophytes, can establish mutually beneficial symbiotic associations with approximately 80% of terrestrial plants, including 90% of crop species [14]. AMF hyphae can explore soil pores inaccessible to root hairs, contributing to the uptake of more water and mineral nutrients than the root system alone; meanwhile, AMF rely on their host plants to complete their lifecycle and obtain organic compounds, including sugars and lipids [15]. This mutualistic symbiosis enhances plant growth and regulates the physiological, morphological, and nutritional processes of host plants, ultimately conferring resistance to a range of abiotic stresses, including high temperature, drought, cold, and salinity [13]. AMF enhance the drought resistance of host plants through different mechanisms including (I) alterations in root and leaf morphology, (II) increased efficiency in water and nutrient absorption, (III) physiological improvements in the signaling pathway, hormone balance, antioxidant systems and photosynthesis, and (IV) regulation of the expression of drought-responsive genes [2,13,16,17]. AMF extraradical hyphae play a critical role in signaling communication between AMF and host plants, due to its ability form complex mycorrhizal networks between neighboring plant roots and rhizosphere soils [18]. AMF regulate diverse signaling pathways to trigger a range of physiological and molecular responses to drought stress; moreover, their symbiosis contributes to plants’ drought tolerance by modulating its drought-responsive genes, encompassing both functional and regulatory genes [18,19]. Functional genes play distinct roles in enhancing plant tolerance to drought, such as aquaporins (AQPs), plasma membrane intrinsic protein (PIP), late embryogenesis abundant (LEA) proteins, and genes encoding enzymes essential for antioxidant defense systems [20]. Conversely, regulatory genes are vital for signaling transduction and function as transcription factors that regulate the expression of downstream genes, thereby coordinating the plant’s response to drought stress [19]. Exploring the molecular mechanism of the Ca^2+^ signaling pathway was an important step in understanding the drought tolerance induced by AMF symbiosis. Through real-time quantitative PCR technology, some literature reports found that the gene expressions of Ca-dependent kinases family members *CsCDPK20* and *CsCDPK22* in *Citrus sinensis* were upregulated by *Funneliformis mosseae* colonization under drought stress [9], *Glomus intraradices* inoculation significantly enhanced the gene expression of *ZmPIP1;1*, *ZmPIP1;3*, *ZmPIP1;4*, *ZmPIP1;6*, *ZmPIP2;2*, *ZmPIP2;4*, *ZmTIP1;2* and *ZmPIP2;5* in *Zea mays* roots under drought stress [21].

Improving drought resistance of crops by AMF inoculation is a well-known agricultural technique, which represents a good strategy for the stability of global food production. The transcriptome analysis explored that *Funneliformis mosseae* inoculation positively affects wheat (*Triticum aestivum* L.) by alleviating drought stress, which is evident through the regulation of wheat genes related to cell wall and membrane elements [22]. Recent transcriptome analysis also disclosed that genes induced by *F. mosseae* in wheat roots under water deficit were enriched in AQPs [13]. Despite the abundance of evidence showing the beneficial effects of AMF symbiosis on maize physiological and molecular responses under drought stress, however, there was still little research on the molecular mechanisms on the Ca^2+^ signaling pathway underlying the drought tolerance of host plants regulated by AMF through high-throughput sequencing and transcriptome analysis. In this study, we hypothesized that AMF inoculation could improve the maize tolerance to drought stress by regulating the gene expression pattern of the Ca^2+^ signaling pathway. To test the hypothesis, a pot experiment was conducted to investigate the impact of *F. mosseae* inoculation on Ca^2+^ flux and concentration in maize shoots and roots under both well-watered (WW) and drought-stressed (DS) conditions, while the differentially expressed maize genes of Ca^2+^ signaling pathway induced by *F. mosseae* inoculation under drought stress were also comprehensively investigated by using high-throughput transcriptomics techniques. Our findings provide novel insights into the molecular mechanism underlying AMF-induced drought tolerance of maize at the transcriptional levels.

## 2. Materials and Methods

### 2.1. Experimental Design and Statistical Analysis

The experiment employed a randomized block design featuring a factorial combination of 2 × 2 (drought stress and AMF inoculation), with each treatment replicated three times, giving a total of 12 plants (one seedling per pot). There were two levels of soil drought treatment (namely, well-watered (WW) and drought-stressed (DS) conditions), and two mycorrhizal inoculation treatments (single inoculation with AMF *Funneliformis mosseae*, and non-AMF inoculation).

### 2.2. Biological Material and Growth Conditions

The AMF strain, *Funneliformis mosseae* BGC XZ02A, was retrieved from the Beijing Academy of Agriculture and Forestry Sciences, Beijing, China. The mycorrhizal inoculum consisted of sandy soil, spores (approximately 45 g^−1^ dry soil), extraradical hyphae, and colonized roots. For each AMF-inoculated treatment, 40 g of AMF inoculum were placed 5 cm below the maize seeds. Each non-AMF treatment received 40 g of sterilized AMF inoculum, along with 30 milliliters of inoculum filtrate, to ensure a similar microbiota excluding AMF.

Seeds of the native maize cultivar (*Zea mays* L. cv. genotype Zhengdan 958) were acquired from a seed market located in Guanlin town, Luoyang, China. Before planting, the seeds were immersed and sterilized in 0.5% K_2_MnO_4_ for 20 min, followed by four thorough rinses with sterile distilled water while agitating. The sterilized seeds were then placed on moist filter paper within Petri dishes and incubated at 28 °C, and sterile purified water was used for seed germination and plant growth. Once the seeds germinated to approximately 2 cm in length, each seedling was transplanted into a plastic container with a diameter of 15 cm and a depth of 15 cm, which was filled with a 2 kg mixture of soil and sand.

The soil (<2 mm) used as culture media was obtained at 0–20 cm depth from topsoil of uncultivated land at Henan University of Science and Technology, China. Sand (<2 mm) was also collected and washed in tap water. The soil and sand were mixed at a volume ratio of 2:1, and then sterilized via steaming at 0.11 Mpa and 121 °C for 2 h. The sterilized soil/sand mixture had the following physicochemical characteristics: a pH of 7.8 (measured at a 1:5 soil/water ratio, *w*/*v*), 15.12 g·kg^−1^ of organic matter, 33.64 mg kg^−1^ of available nitrogen, 10.78 mg kg^−1^ of Olsen phosphorus, and 79.83 mg kg^−1^ of available potassium.

Between April and July 2021, the seedlings were grown in a phytochamber with a 12-h day/12 h night cycle, maintained at temperatures of 36 °C during the day and 20 °C at night, and with air humidity ranging from 70% to 90%. The average photosynthetic photon flux density was set at 1000 μmol m^−2^ s^−1^. To promote the establishment of a symbiotic relationship between AMF and the maize roots, the seedlings were watered twice weekly for two months with 100 mL of a modified Hoagland nutrient solution, enriched with 25 μM phosphate (Pi), to encourage mycorrhizal colonization. Two months after transplanting the seedlings, the DS treatment was initiated. The drought-stressed phase began in July 2021 and lasted for one month. During this period, half of the pots were kept under well-watered (WW) conditions, with a soil moisture level maintained at 75% field capacity (equivalent to a water potential of −0.12 MPa). The other half of the pots were subjected to drought stress (DS), with soil moisture maintained at 45% field capacity (equivalent to a water potential of −0.66 MPa). To ensure consistent soil moisture levels, the pots were regularly weighed, and fresh distilled water was added every three days to compensate for any water loss.

### 2.3. Detection of Ca^2+^ Fluxes and Contents in Maize Leaves and Roots

Ca^2+^ fluxes were measured utilizing the non-invasive micro-test technique (NMT-YG-100, Younger USA LLC, Amherst, MA, USA). Following a one-month treatment period, the root tips (1–2 cm) of maize plants and the middle segment of the third leaves were selected for steady Ca^2+^ flux analysis. A specialized selective micro-electrode (model MicroC-300, Unisense Microsensors, Aarhus, Denmark) for Ca^2+^ was used to measure the ion levels, with continuous recording of ion flux captured for a duration of 300 s per sample. Subsequently, the maize leaves and roots were subjected to drying at 75 °C for 24 h in an oven, after which they were ground into a fine powder. Ca^2+^ ions were extracted using a microwave digestion instrument (Bayue BYWB-40, Changsha, China) with H_2_O_2_-H_2_SO_4_. The extracted Ca^2+^ ions were then assayed using an atomic absorption spectrophotometer (PerkinElmer PE800, Waltham, MA, USA) to determine their content.

### 2.4. Total RNA Extraction, Library Preparation, and Illumina Hiseq Sequencing

The fresh maize roots and leaves were, respectively, immersed in liquid nitrogen and ground in mortars. Total RNA was extracted from entire maize seedlings using the TRIzol^®^ Reagent (Invitrogen, Carlsbad, CA, USA), following the manufacturer’s protocol. High-quality RNA was checked on a gel to see the intact ribosomal RNA bands; RNA integrity was further assessed using the Agilent TapeStation system (Santa Clara, CA, USA), and only samples with RIN (RNA Integrity Number) ≥ 7.0 were retained for sequencing. Prior to Illumina sequencing, the extracted RNA samples were evaluated using a NanoDrop spectrophotometer ND-2000 (Thermo Fisher Scientific Inc., Waltham, MA, USA). Only high-quality RNA samples (≥200 ng/μL RNA concentration, and 1.8–2.2 OD260/280) were selected and analyzed for constructing the sequencing library, and RNA-Seq analysis was conducted by using the Illumina NovaSeq 6000 platform at BIOZERON Co., Ltd. (Shanghai, China).

After total RNA extraction, mRNA was enriched using poly—T oligos—coated magnetic beads targeting eukaryotic mRNA poly—A tails. The mRNA was fragmented into ~200–300 bp segments with fragmentation buffer at high temperature. First-strand cDNA was synthesized from fragmented mRNA using random hexamer primers and reverse transcriptase. Second-strand cDNA was then made with DNA polymerase I, RNase H, and dNTPs. The double-stranded cDNA was end-repaired for blunt ends, followed by 3′ A-tailing. Illumina adapters were ligated, and the adapter-ligated libraries were size-selected via agarose gel electrophoresis. PCR amplification enriched the libraries and added index sequences for multiplexing.

The libraries’ quality and quantity were assessed with a Qubit fluorometer and Agilent 2100 Bioanalyzer (Santa Clara, CA, USA). Validated libraries were diluted and denatured into single-stranded DNA molecules, loaded onto the Illumina HiSeq flow cell. Bridge amplification generated millions of identical clusters on the flow cell surface. Paired-end sequencing simultaneously sequenced both ends of each DNA fragment, yielding high-throughput, accurate reads.

A total of 24 cDNA libraries were constructed and sequenced through eight treatments (CKWR, CKDR, CKWL, CKDL, AWR, ADR, AWL, and ADL) with triplicate samples for each: A: *F*. *mosseae* inoculation; CK: non-mycorrhizal inoculation; D: drought stress; W: well-watered; R: roots; and L: leaves). Reference genome and gene annotation files were downloaded from the website (http://plants.ensembl.org/Zea_mays/Info/Index, accessed on 23 October 2023), and the reference genome (B73_RefGen_v4) was used in our study. All clean reads were mapped to the maize genome reference sequences by SOAPaligner/soap2. The calculation of gene expression uses RPKM method (reads per kb per million reads). The raw reads were available in the Sequence Repository Archive (SRA) of the NCBI database under the accession PRJNA1077452.

### 2.5. De Novo Assembly, Functional Annotation, and Identification of Differential Expression Genes

To control the quality of original data prior to transcript assembly, the Cutadapt v2.10 software was utilized to remove adapter sequences, and the Trinity v2.10.0 software was employed to assemble the filtered data and construct the unigene set of sequences [23]. The longest sequence was used as the reference transcript sequence (Unigene) for subsequent analysis. All obtained unigenes were functionally annotated by comparing them against various databases using BLASTX (https://blast.ncbi.nlm.nih.gov/Blast.cgi, accessed on 3 December 2023) with an E-value cutoff of 1 × 10^−5^. These databases included NR (NCBI’s non-redundant protein sequences), KEGG (Kyoto Encyclopedia of Genes and Genomes), GO (Gene Ontology), and COG (Swiss-Prot, Clusters of Orthologous Groups of proteins) at an E-value cut-off of 1 × 10^−5^. To identify differential expression genes (DEGs) between two different samples, RSEM software (version 1.2.15) was used for quantifying transcript abundances on Unigene. Gene differential expression analysis was conducted using the R statistical package software EdgeR (https://bioconductor.org/packages/release/bioc/html/edgeR.html, accessed on 12 February 2024). The expression levels of each transcript were quantified using reads per kilobase of exon per million mapped reads (RPKM). To identify genes specifically modulated by AMF inoculation and/or drought stress in maize shoots and roots, transcripts with a *p*-value < 0.05 and a |log2FoldChange| ≥ 2 were considered as differentially expressed genes (DEGs) among the three groups (drought conditions (DS and WW), AMF inoculation (*F. mosseae* inoculation and non-mycorrhizal inoculation) and tissue (maize shoots and roots)).

### 2.6. Functional Enrichment of DEGS and Identification of Transcription Factor

To better understand the functional categories of DEGs regulated by drought stress and *F. mosseae* inoculation in maize seedlings, GO and KEGG enrichment analyses were conducted for DEGs in various comparisons, including drought vs. tissue, drought vs. AMF, tissue vs. AMF, and drought vs. AMF vs. tissue. These analyses were performed to identify DEGs that were significantly enriched in GO terms and KEGG metabolic pathways, using Goatools (available at https://github.com/tanghaibao/Goatools, accessed on 23 February 2024) and KOBAS (available at http://bioinfo.org/kobas, accessed on 25 February 2024), respectively, with a *p*-value ≤ 0.05 against the whole-transcriptome background. Transcription factors (TFs) were identified using the Plant Transcription Factor Database v5.0 (available at https://planttfdb.gao-lab.org/, accessed on 27 February 2024).

### 2.7. Co-Expression Network Analysis

Gene co-expression network analysis was performed for calcineurin B-like (CBL) proteins, CBL-interacting protein kinases (CIPKs), mitogen-activated protein kinases (MAPKs), and calcium-dependent protein kinases (CDPKs) in maize. The Pearson correlation coefficient (PCC) was calculated using SPSS 25 software (SPSS Statistics, IBM, New York, NY, USA). Gene pairs of CBLs, CIPKs, MAPKs, and CDPKs with a PCC *p*-value < 0.05 were collected and visualized to construct co-expression networks using Cytoscape 3.10.1 software [24]. Additionally, a co-expression network for transcription factors (TFs) and Ca^2+^ signaling pathway-related genes in maize was also generated and visualized using the same method. Only TFs with a sub-network consisting of at least four genes were considered for further analysis.

## 3. Results

### 3.1. Ca^2+^ Fluxes and Contents in Maize Roots and Leaves

**Figure 1:**
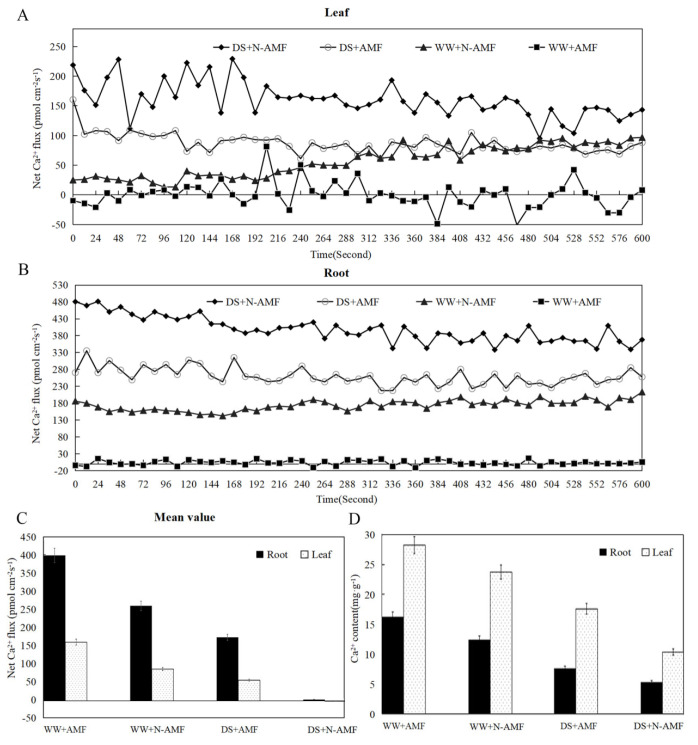
Effects of drought stress and *F. mosseae* on the net Ca^2^⁺ fluxes and Ca^2^⁺ content in maize roots and leaves under well-watered (WW) and drought-stressed (DS) conditions. The flux rates of Ca^2+^ were measured for 600s. (**A**) The leaf of net Ca^2+^ fluxes; (**B**) the root of net Ca^2+^ fluxes; (**C**) the mean value of net Ca^2+^ fluxes in maize roots and leaves; (**D**) the Ca^2+^ content in maize roots and leaves. N-AMF: non-mycorrhizal inoculation, AMF: *Funneliformis mosseae* inoculation. Values are means ± SD, *n* = 3.

Investigations into ion kinetics revealed the net fluxes of Ca^2+^ in maize roots and leaves (Figure 1). Drought stress decreased the net fluxes of Ca^2+^ in both maize roots (Figure 1A) and leaves (Figure 1B); conversely, *F. mosseae* inoculation enhanced these fluxes of Ca^2+^ in both tissues. Under WW conditions, the mean net Ca^2+^ flux rates in roots and leaves of non-inoculated seedlings were 259.93 and 87.03 pmol/(cm^−2^·s), respectively. However, these rates were significantly higher in *F. mosseae*-inoculated seedlings, reaching 398.36 pmol/(cm^−2^·s) in the roots and 160.72 pmol/(cm^−2^·s) in the leaves (Figure 1C). Under DS conditions, the mean net Ca^2+^ flux rates in the roots and leaves of non-inoculated seedlings dropped to 3.63 and −0.11 pmol/(cm^−2^·s), respectively. In contrast, *F. mosseae*-inoculated seedlings exhibited substantially higher rates of 174 pmol/(cm^−2^·s) in the roots and 56.41 pmol/(cm^−2^·s) in the leaves (Figure 1C). Additionally, the Ca^2+^ contents in maize leaves were consistently higher than those in the roots. Drought stress decreased the Ca^2+^ content in maize roots and leaves, but *F. mosseae* inoculation enhanced the Ca^2+^ contents in both tissues (Figure 1D).

### 3.2. Transcriptome Sequencing and Gene Annotation

To better understand the drought tolerance mechanism in mycorrhizal maize at the molecular level, the differential gene expression of maize shoots and roots in response to *F. mosseae* inoculation under drought stress was investigated by the method of RNA-seq. The clean data of each sample were above 6 Gb, with the average base error rate remaining below 0.1%. After removing low-quality raw reads (Q-value < 20) and trimming adaptor sequences, the percentage of Q20 and Q30 bases attained values greater than 97.69% and 93.78%, respectively, and the GC content exceeded 51.53% (Table 1A). To gain insights into the functional annotation of expressed unigenes (transcripts), we performed comprehensive annotation against six major biological databases. A total of 35,283 (138,412) unigenes were mapped to GO, 16,514 (80,595) to KEGG, 37,828 (149,246) toCOG, 42,327 (155,685) to NR, 28,570 (125,447) to Swiss-Prot, and 29,227 (116,275) to Pfam. Analysis across all six databases revealed distinct totals for molecular features: 42,446 annotated expressed unigenes, 155,829 expressed transcripts, 48,662 total unigenes, and 174,675 total transcripts were identified (Table 1B). When considering additional quality control parameters, these figures increased to 46,725 annotated expressed unigenes, 160,987 expressed transcripts, 56,933 total unigenes, and 183,919 total transcripts, respectively (Table 1B).

### 3.3. Identification of Differential Expression Genes (DEGs) Related to F. mosseae Inoculation and Drought Stress in Maize Roots and Leaves

**Figure 2:**
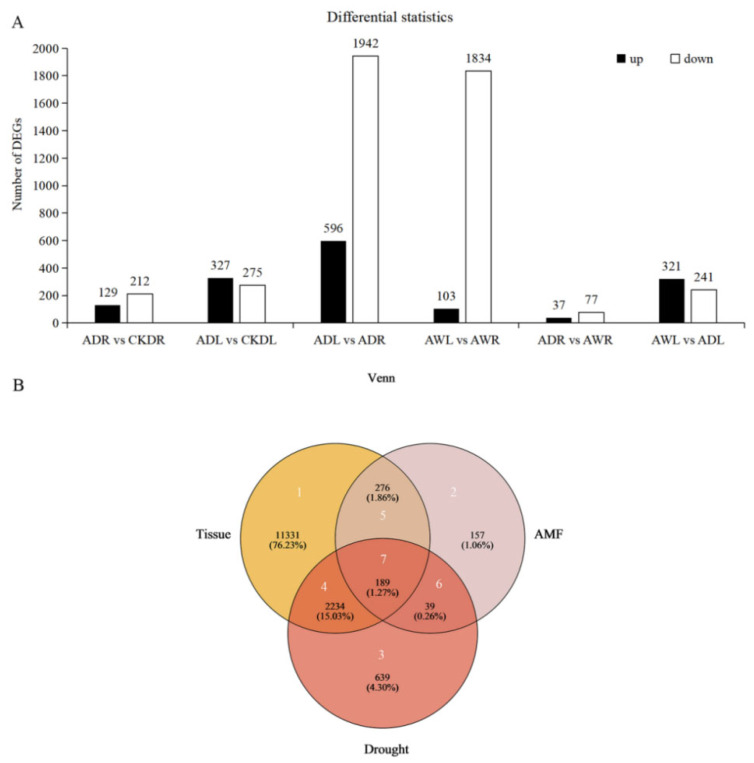
Differentially expressed genes (DEGs) regulated by tissue, AMF symbiosis, and drought stress. (**A**) The number of DEGs related to AMF inoculation and drought stress was identified by pairwise comparison of the expression levels of maize roots and leaves. A: *F. mosseae* inoculation, CK: non-mycorrhizal inoculation, D: drought stress, W: well-watered, R: roots, L: leaves. (**B**) Venn diagram showing the overlap of DEG among the three treatment groups. White number: region number; black number: number of DEGs; black number inside parenthesis, percentage of the total DEGs.

Through a pairwise comparison of their expression levels in maize roots and leaves, the identification of differentially expressed genes (DEGs) associated with *F. mosseae* inoculation and drought stress was obtained. This analysis provided novel perspectives into the role of AMF symbiosis with maize under drought stress (Figure 2A). Under DS conditions, *F. mosseae* inoculation upregulated 129 DEGs and downregulated 212 DEGs in maize roots (ADR), meanwhile *F. mosseae* inoculation induced a significantly higher number of upregulated DEGs (327) than downregulated DEGs (275) in maize leaves (ADL). Notably, under DS conditions, and when inoculated with *F. mosseae*, the number of downregulated DEGs (1942) was more than three times higher than the number of upregulated DEGs (596) in the comparison between leaves (ADL) and roots (ADR). However, under WW conditions, the leaves of *F. mosseae*-inoculated maize (AWL) exhibited a remarkable imbalance, with downregulated DEGs (1834) almost 13 times higher than the upregulated DEGs (103), compared with the roots of *F. mosseae*-inoculated maize (AWR). The upregulated and downregulated DEGs of ADR were 37 and 77, respectively, compared with AWR. Similarly, there were 321 upregulated and 241 downregulated DEGs of AWL, compared with ADL.

DEGs influenced by three factors, water conditions (drought stress (DS) versus well-watered (WW) conditions), AMF inoculation (*F. mosseae* inoculation versus non-mycorrhizal inoculation) and tissue type (maize leaves versus roots), were identified by using cutoffs of |log_2_ (fold-change)| > 1 and FDR < 0.05. A total of 14,865 differentially expressed genes (DEGs) were identified across the three treatment groups (Figure 2B). To further investigate the relationships and variations among these DEGs, a Venn diagram was constructed, categorizing them into seven distinct regions (Figure 2B). In region 1, 11,331 DEGs (76.23%) displayed differential expression between maize leaves and roots, but were unaffected by AMF inoculation or drought stress. Region 2 comprised 157 DEGs (1.06%) that were regulated by AMF symbiosis, without exhibiting tissue-specific or drought-responsive patterns. Region 3 contained 639 DEGs (4.30%), regulated by drought stress but independent of tissue preference or AMF response. Region 4 encompassed 2234 genes (15.03%) that exhibited drought-responsive and tissue-preferred expression patterns, yet remained unresponsive to AMF symbiosis. In region 5, 276 DEGs (1.86%) indicated tissue-preferred and AMF-responsive expression patterns, but were unaffected by drought stress. Region 6 had 39 DEGs (0.26%) that were modulated by both AMF symbiosis and drought stress, but did not show similar expression levels in both maize leaves and roots. Lastly, region 7 comprised 189 DEGs (1.27%) that responded not only to AMF symbiosis and drought stress but also exhibited preferential expression in either leaves or roots (Figure 2B).

### 3.4. Functional Annotation of DEGs in Maize Leaves and Roots Induced by F. mosseae Inoculation Under Drought Stress

To gain insights into the molecular mechanisms underlying the effect of *F. mosseae* inoculation on maize under drought stress, the functional categories of regions 4, 5, 6, and 7 (Figure 2B) were identified. The DEGs from these regions were subjected to GO and KEGG annotation analyses. These DEGs were assigned to at least one GO term, categorizing them based on their biological processes (BP), cellular components (CC), and molecular functions (MF). Similarly, they were classified into various KEGG metabolic pathway categories, including metabolism (M), environmental information processing (EIP), organismal systems (OS), cellular processes (CP), and genetic information processing (GIP).

**Figure 3:**
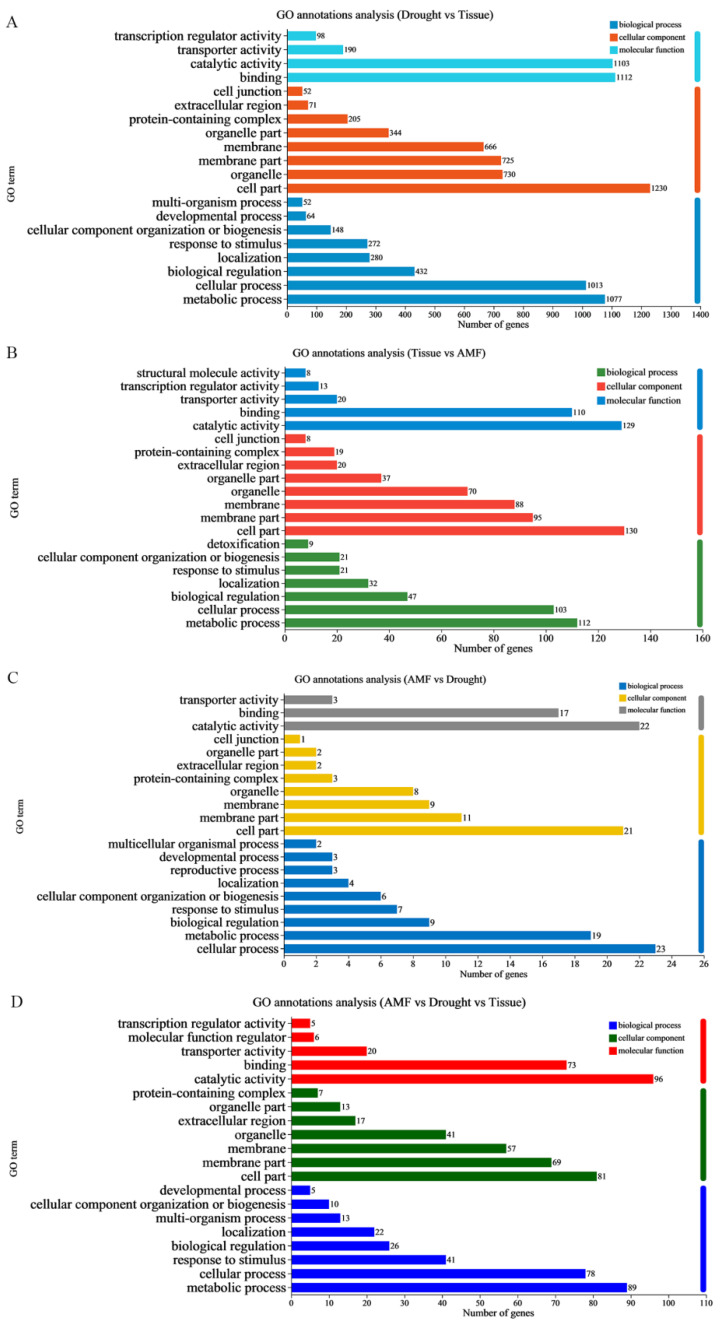
GO annotations analysis for DEGs A histogram was used to visualize the Gene Ontology (GO) annotations analysis of DEGs. GO terms from the cellular component (CC), biological process (BP), and molecular function (MF) categories were plotted. (**A**) GO annotations in Drought versus Tissue. (**B**) GO annotations in Tissue versus AMF. (**C**) GO annotations in AMF versus Drought. (**D**) GO annotations in AMF versus Drought versus Tissue.

**Figure 4:**
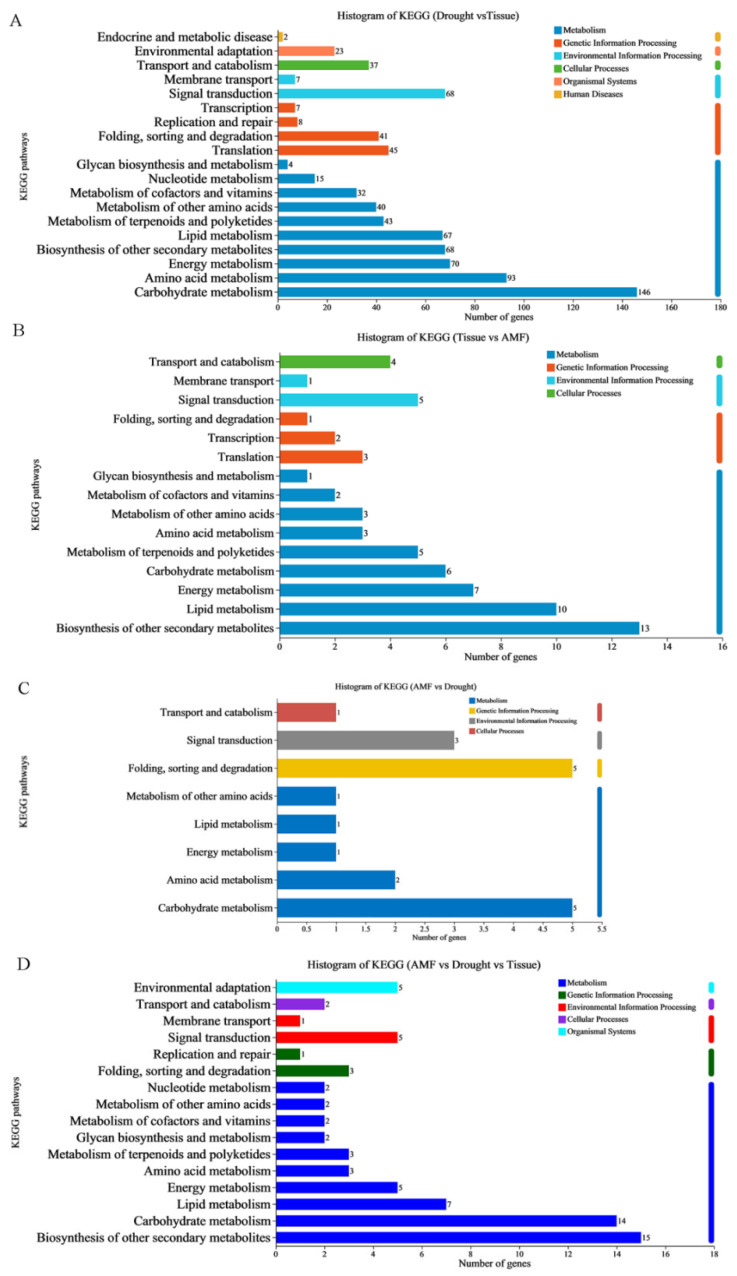
KEGG annotation analysis for DEGs. A histogram was used to visualize the Kyoto Encyclopedia of Genes and Genomes (KEGG) annotation analysis of DEGs. KEGG terms from the metabolism (M), environmental information processing (EIP), organismal systems (OS), cellular processes (CP), and genetic information processing (GIP) groups were plotted. (**A**) KEGG annotations in Drought versus Tissue. (**B**) KEGG annotations in Tissue versus AMF. (**C**) KEGG annotations in AMF versus Drought. (**D**) KEGG annotations in AMF versus Drought versus Tissue.

In region 4, GO annotations revealed that DEGs exhibiting drought-responsive and tissue-preferred expression patterns were primarily annotated with ‘metabolic process’ (1077), ‘cellular process’ (1013), and ‘biological regulation’ (432) within the BP group (Figure 3A). Among the CC group, terms such as ‘cell part’ (1230), ‘organelle’ (730), ‘membrane part’ (725), and ‘membrane’ (666) were most prominent. In the MF group, ‘binding’ (1112) and ‘catalytic activity’ (1103) were the predominant terms. As expected, KEGG annotations in region 4 indicated that DEGs were mainly associated with ‘translation’ (45) and ‘folding, sorting, and degradation’ (41) in the GIP group, ‘transport and catabolism’ (37) in the CP group, ‘environmental adaptation’ (23) in the OS group, and ‘signal transduction’ (68) in the EIP group. In the M group, DEGs were primarily annotated with ‘carbohydrate metabolism’ (146), ‘amino acid metabolism’ (93), ‘energy metabolism’ (70), ‘biosynthesis of other secondary metabolites’ (68), and ‘lipid metabolism’ (67) (Figure 4A).

In region 5, DEGs displaying AMF-responsive and tissue-specific expression patterns were predominantly annotated within the BP group with terms like ‘metabolic process’ (112), ‘cellular process’ (103), and ‘biological regulation’ (47) (Figure 3B). Within the CC group, the most represented terms were ‘cell part’ (130), ‘membrane part’ (95), ‘membrane’ (88), and ‘organelle’ (70). Among the MF group, ‘catalytic activity’ (129) and ‘binding’ (110) emerged as the most frequently matched terms. KEGG pathway mapping in region 5 revealed that DEGs were primarily associated with ‘biosynthesis of other secondary metabolites’ (13), and ‘lipid metabolism’ (10) (Figure 4B).

In region 6, DEGs exhibiting drought-responsive and tissue-specific expression patterns were primarily annotated within the BP group with terms such as ‘cellular process’ (23), ‘metabolic process’ (19), and ‘biological regulation’ (9) (Figure 3C). Among the CC group, the most prevalent annotations were ‘cell part’ (21), and ‘membrane part’ (11). Within the MF group, ‘catalytic activity’ (22) and ‘binding’ (17) emerged as the dominant matched terms (Figure 4C).

In region 7, DEGs exhibiting tissue-preferred expression patterns in response to both AMF symbiosis and drought likely play a crucial role in AMF-induced drought tolerance. Within the BP group, the most represented annotations were ‘metabolic process’ (89), ‘cellular process’ (78), and ‘response to stimulus’ (41) (Figure 3D). In the CC group, the prevalent terms were ‘cell part’ (81), ‘membrane part’ (69), ‘membrane’ (57), and ‘organelle’ (41). Among the MF group, ‘catalytic activity’ (96) and ‘binding’ (73) were the primary matched terms. KEGG annotations in region 7 revealed that DEGs were mainly annotated with ‘biosynthesis of other secondary metabolites’ (15), and ‘carbohydrate metabolism’ (14) (Figure 4D) in the M group.

### 3.5. Functional Enrichment of DEGs in Maize Shoots and Roots Induced by F. mosseae Inoculation Under Drought Stress

**Figure 5:**
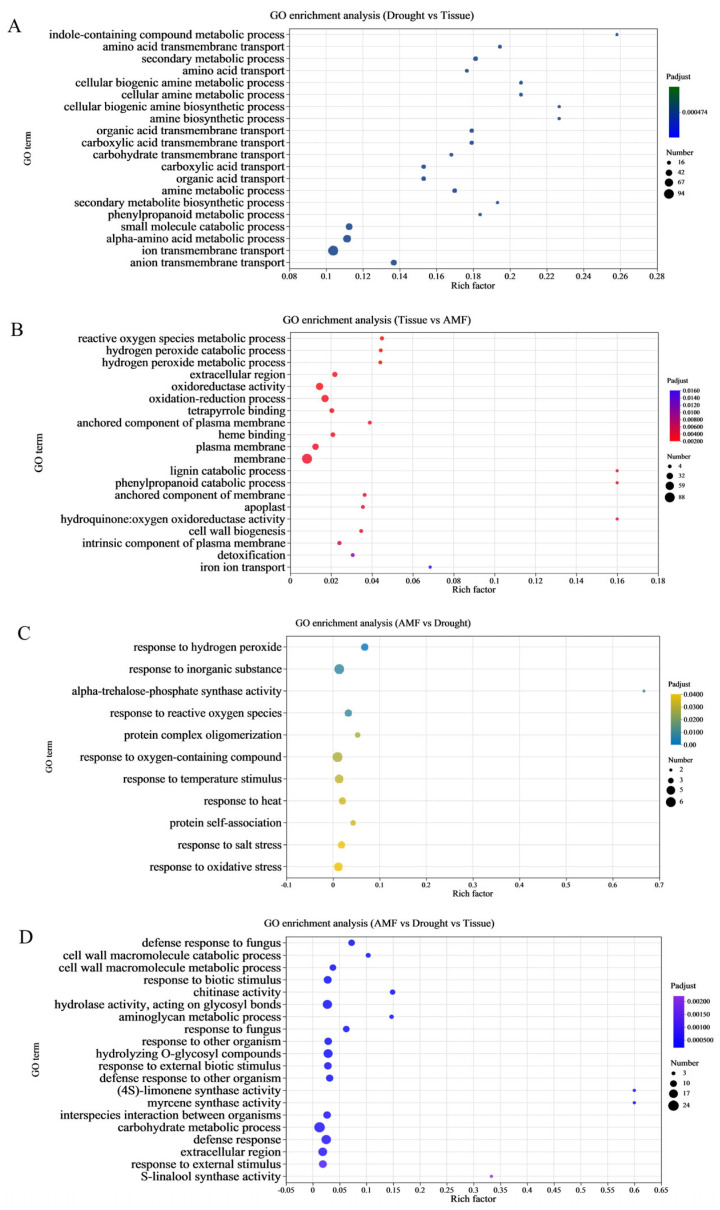
GO enrichment analysis for DEGs. A bubble plot was used to visualize the Gene Ontology (GO) enrichment analysis of DEGs. (**A**) GO enrichment in Drought versus Tissue. (**B**) GO enrichment in Tissue versus AMF. (**C**) GO enrichment in AMF versus Drought. (**D**) GO enrichment in AMF versus Drought versus Tissue.

**Figure 6:**
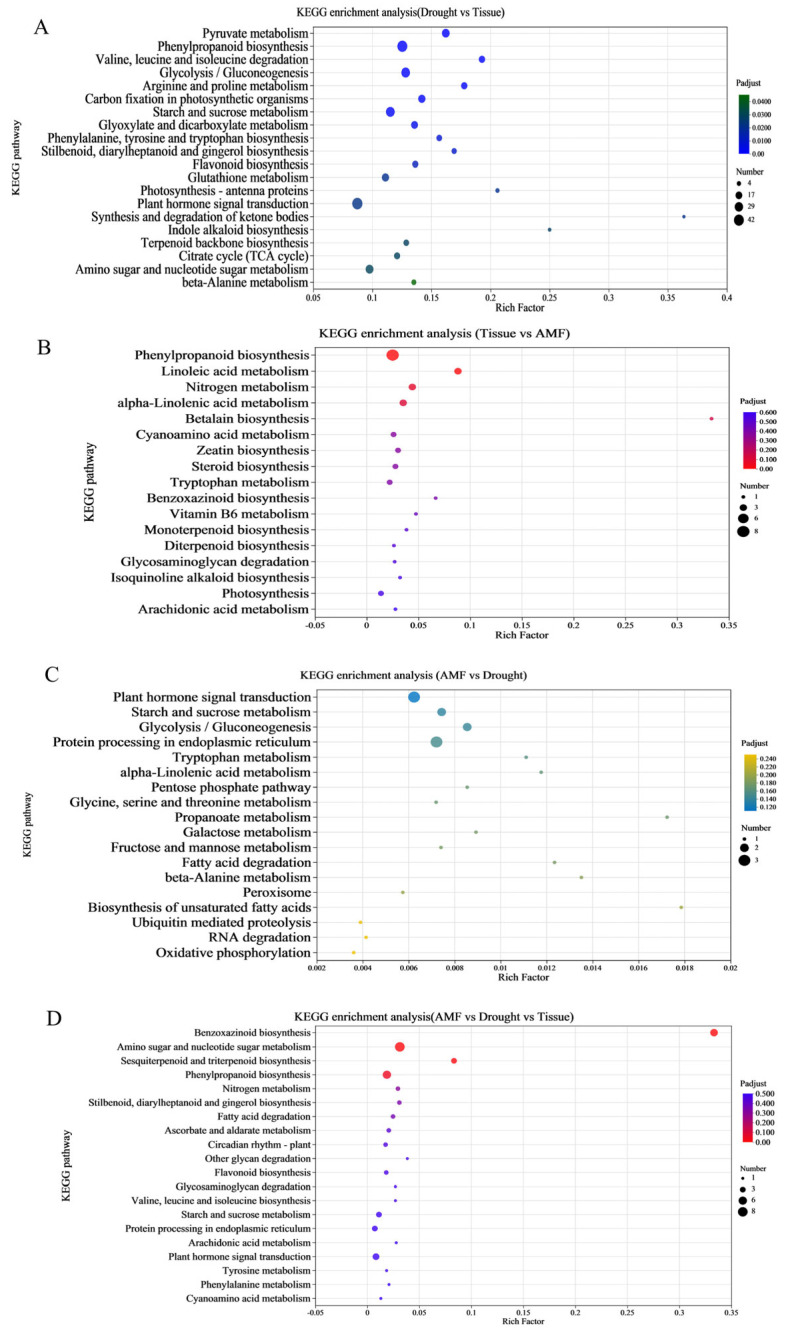
KEGG enrichment analysis for DEGs. A bubble plot was used to visualize the Kyoto Encyclopedia of Genes and Genomes (KEGG) enrichment analysis of DEGs. (**A**) KEGG enrichment in Drought versus Tissue. (**B**) KEGG enrichment in Tissue versus AMF. (**C**) KEGG enrichment in AMF versus Drought. (**D**) KEGG enrichment in AMF versus Drought versus Tissue.

To identify the functional categories of regions 4, 5, 6, and 7 (Figure 2B), the DEGs from these regions were subjected to GO and KEGG enrichment analyses. In region 4, GO enrichment analysis was used for elucidating the functions of DEGs, categorizing them into biological process, cellular component, and molecular function (Figure 5). The top 20 most significantly enriched GO terms (*p*-value ≤ 0.05) among the DEGs. In region 4 of the drought vs. tissue comparison, the GO terms ‘terpene synthase activity’, ‘indole-containing compound metabolic process’, and ‘amino acid transmembrane transport’ emerged as the most significantly enriched. Furthermore, a larger proportion of enriched GO terms belonged to ‘molecular_function’ (1728) and ‘biological_process’ (1572) (Figure 5A). Based on the KEGG pathway analysis, 1032 exclusive DEGs were enriched, with the top 20 most significantly enriched KEGG pathways for DEGs (*p*-value ≤ 0.05) displayed in Figure 5. These pathways were organized into six primary KEGG categories. In region 4 of the drought vs. tissue comparison, the top pathway of the first category, encompassing the highest number of DEGs, was ‘plant hormone signal transduction’ (42), followed by ‘phenylpropanoid biosynthesis’ (40), and ‘starch and sucrose metabolism’ (31) (Figure 6A). Among these, ‘phenylpropanoid biosynthesis’, ‘pyruvate metabolism’, and ‘valine, leucine and isoleucine degradation’ emerged as the most significantly enriched pathways in the first category.

In region 5 of the tissue vs. AMF comparison, the GO terms ‘extracellular region’, ‘reactive oxygen species metabolic process’, and ‘hydrogen peroxide catabolic process’ emerged as the most enriched. Furthermore, the largest number of enriched GO terms related to the categories ‘cellular anatomical entity’ (177) and ‘catalytic activity’ (129) (Figure 5B). Additionally, in region 5 of the tissue vs. AMF comparison, KEGG pathway analysis revealed that 96 exclusive DEGs were enriched. The pathways ‘phenylpropanoid biosynthesis’ (8 DEGs), and ‘plant hormone signal transduction’ (4 DEGs) topped the list in terms of the number of involved DEGs (Figure 6B). Among these, ‘phenylpropanoid biosynthesis’, ‘linoleic acid metabolism’, and ‘nitrogen metabolism’ emerged as the most significantly enriched pathways within the first category.

In region 6, comparisons between AMF and drought conditions, the most significantly enriched GO terms were ‘response to hydrogen peroxide’, ‘alpha, alpha-trehalose-phosphate synthase (UDP-forming) activity’, and ‘response to inorganic substance’. Among the enriched GO terms, a larger number belonged to the categories ‘biological_process’ (33) and ‘small molecule metabolic process’ (7) (Figure 5C). The results of the KEGG pathway analysis revealed that 19 unique DEGs in region 6 were enriched. The top pathway of the first category involving the largest number of DEGs were ‘Oxidative phosphorylation’ (3), ‘RNA degradation’ (3), and ‘Ubiquitin mediated proteolysis’ (2) (Figure 6C). Among these, the most significantly enriched pathways in the first category were ‘Protein processing in endoplasmic reticulum’, ‘Plant hormone signal transduction’, and ‘Glycolysis/Gluconeogenesis’.

In region 7 of AMF vs. drought vs. tissue comparison, the most significantly enriched GO terms were ‘chitinase activity’, ‘defense response to fungus’, and ‘cell wall macromolecule catabolic process’. Notably, a substantial number of enriched GO terms belonged to the categories of ‘biological process’ (140) and ‘catalytic activity’ (96) (Figure 5D). The results of the KEGG pathway analysis revealed that 87 unique DEGs in region 7 were enriched. The KEGG pathways with the highest number of DEGs in the first category included ‘amino sugar and nucleotide sugar metabolism’ (8), ‘phenylpropanoid biosynthesis’ (6), and ‘benzoxazinoid biosynthesis’ (5) (Figure 6D). Among these, the most significant enriched pathways in the first category were ‘benzoxazinoid biosynthesis’, ‘amino sugar and nucleotide sugar metabolism’, and ‘sesquiterpenoid and triterpenoid biosynthesis’. 

### 3.6. Expression Patterns of DEGs Involved in Calcineurin B-like (CBL) Protein, CBL-Interacting Protein Kinase (CIPK), Mitogen Activated Protein Kinase (MAPK), and Calcium Dependent Protein Kinase (CDPK) Regulated by F. mosseae Inoculation Under Drought Stress

**Figure 7:**
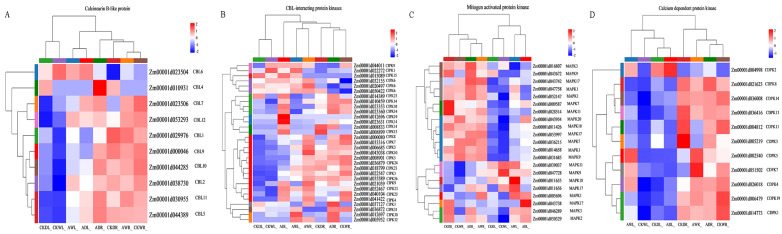
Heatmap of the expression patterns of DEGs of maize involved in calcineurin B-like protein (CBL, (**A**)), calcineurin interacting protein kinase (CIPK, (**B**)), mitogen activated protein kinase (MAPK, (**C**)) and calcium dependent protein kinase (CDPK, (**D**)) regulated by AMF inoculation under drought stress. Each column represents a sample, each row represents a gene, and the colors in the graph represent the expression level of the gene after standardization in each sample.

To better comprehend the molecular mechanisms underlying the alleviation of drought stress through AMF symbiosis, we investigated the expression patterns of DEGs related to the Ca^2+^ signaling pathway, including genes encoding CBLs, CIPKs, MAPKs, and CDPKs. Cluster analysis of gene sets revealed that ten CBL genes showed differential expression patterns in maize roots and leaves (Figure 7A). Under WW conditions, *F. mosseae* inoculation downregulated the gene expression of *ZmCBL2*, *7*, *9*, *10*, and *12*, and upregulated *ZmCBL1*, *5*, *6*, and *11* in maize roots, *F. mosseae* inoculation also downregulated *ZmCBL6*, but upregulated *ZmCBL1*, *2*, *5*, *7*, *9*, *10*, *11*, and *12* in maize leaves. However, under DS conditions, *F. mosseae* inoculation induced *ZmCBL4*, *5*, *6*, *7*, *10*, *11*, and *12*, and repressed *ZmCBL1*, *2*, and *9* in maize roots, while *F. mosseae* inoculation also induced the gene expression of *ZmCBL2*, *5*, *6*, *7*, *9*, *10*, *11*, and *12*, and repressed the expression of *ZmCBL1*, and *4* in maize leaves. Furthermore, under drought stress and *F. mosseae* inoculation conditions, the maize leaves exhibited upregulation in *ZmCBL6* expression and downregulation in *ZmCBL1*, *2*, *4*, *5*, *7*, *9*, *10*, *11*, and *12*, compared to the maize roots.

Cluster analysis revealed that thirty-one CIPK genes were expressed in maize seedlings, displaying diverse expression patterns (Figure 7B). Under WW conditions, *F. mosseae* inoculation downregulated the expression of *ZmCIPK1*, *4*, *5*, *7*, *8*, *24*, *30*, and *31*, and upregulated *ZmCIPK6*, *26*, and *32* in maize roots, and *F. mosseae* inoculation also downregulated *ZmCIPK1* and *9*, but upregulated *ZmCIPK4*, *5*, *7*, *8*, *15*, *24*, *26*, *30*, *31*, and *32* in maize leaves. However, under DS conditions, *F. mosseae* inoculation induced the expression of *ZmCIPK1*, *3*, *5*, *7*, *8*, *10*, *30*, *31*, and *32*, and repressed *ZmCIPK4*, *6*, *9*, *13*, *14*, and *23* in maize roots, while it also induced the expression of *ZmCIPK1*, *4*, *7*, *8*, *9*, *10*, *13*, *14*, *15*, *19*, *23*, *24*, *26*, and *30*, and repressed the expression of *ZmCIPK3* and *31* in maize leaves. Under drought stress and *F. mosseae* inoculation conditions, *ZmCIPK1*, *4*, *6*, *9*, *13*, *15* and *19* were upregulated expression, whereas *ZmCIPK3*, *5*, *7*, *8*, *24*, *26*, *30*, *31* and *32* were downregulated in the maize leaves, compared to the maize roots.

Cluster analysis of gene sets revealed that twenty-one MAPK genes were expressed in maize roots and leaves, exhibiting distinct differential expression patterns (Figure 7C). Under WW conditions, *F. mosseae* inoculation downregulated the expression of *ZmMAPK1*, *7*, *9 and 20*, and upregulated *ZmMAPK1*, *2*, *3*, *9*, *11*, *17* and *18* in maize roots, it also downregulated *ZmMAPK1*, *3*, *7*, *9*, *10*, *17* and *20*, but upregulated *ZmMAPK1*, *2*, *3*, *7*, *17* and *18* in maize leaves. However, under DS conditions, *F. mosseae* inoculation induced *ZmMAPK1*, *2*, *3*, *7*, *10*, *11* and *17*, and repressed the expression of *ZmMAPK3*, *7*, *9* and *11* in maize roots, while it also induced the expression of *ZmMAPK1*, *2*, *3*, *7*, *9*, *10*, *17* and 20, and repressed the expression of *ZmMAPK1*, *9*, *11* and *18* in maize leaves.

Cluster analysis of gene sets revealed that eleven CDPK genes were expressed in maize seedlings, exhibiting distinct expression patterns (Figure 7D). Under WW conditions, *F. mosseae* inoculation downregulated the expression of *ZmCDPK3*, *4*, *11*, *13*, *14* and *19*, and upregulated *ZmCDPK2*, *5* and *7* in maize roots; *F. mosseae* inoculation also upregulated *ZmCDPK1*, *2*, *3*, *4*, *5*, *7*, *8*, *13*, *14* and *19* in maize leaves. However, under DS conditions, *F. mosseae* inoculation induced *ZmCDPK2*, *4*, *5*, and *7*, and repressed *ZmCDPK1*, *3*, *11*, and *13* in maize roots, while *F. mosseae* inoculation also induced the expression of *ZmCDPK2*, *4*, *13*, and *19*, and repressed the expression of *ZmCDPK1*, *3*, *7*, *8* and *11* in maize leaves. Additionally, under DS and *F. mosseae* inoculation conditions, *ZmCDPK2* expression was upregulated, and *ZmCDPK1*, *3*, *4*, *5*, *7*, *8*, *11*, *13*, *14* and *19* were downregulated in the maize leaves, compared to the maize roots.

### 3.7. Identification and Co-Expression Network Analysis of Transcription Factors (Tfs)

**Figure 8:**
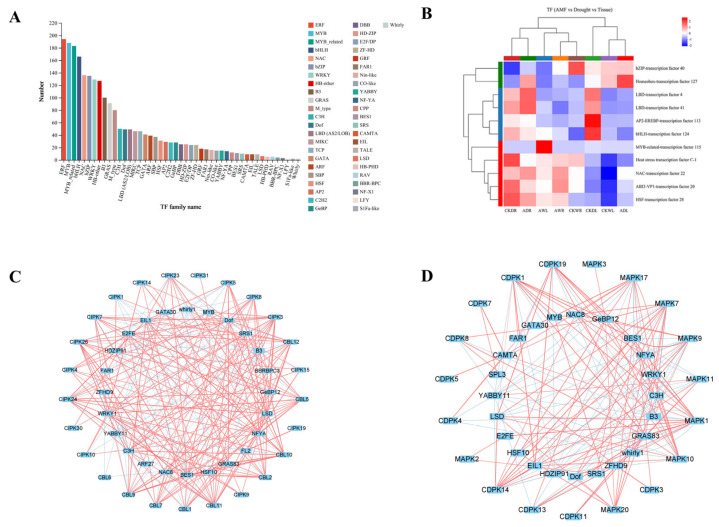
Prediction and co-expression network analysis of transcription factors in maize. (**A**) Prediction of transcription factor family. (**B**) Heatmap of the expression patterns of the 11 TFs in AMF versus Drought versus Tissue. (**C**) Co-expression network analyses contained 25 CBL-CIPK genes and 25 TFs. (**D**) Co-expression network analyses contained 19 CDPK-MAPK genes and 23 TFs. All the PCC of co-expression gene pairs were significant at *p* < 0.05, red lines indicated positive correlations (PCC ≥ 0.6), and blue lines indicated negative correlations (PCC ≤ −0.6), respectively.

Constructing co-expression networks based on the correlations among expression levels was a valuable method for investigating potential gene interactions and regulatory relationships. In this study, a total of 2284 TFs were predicted and identified in transcriptome sequencing data (Figure 8A), and TFs with different expression patterns in response to drought stress and AMF inoculation were considered for further analysis. The ERF subfamily was found to be the largest number of TF family, with 194 genes in total, and there were more MYB and bHLH TF families, with 188 and 166 genes, respectively; in addition, NF-X1, LFY, S1Fa-like, and Whirly TF families were also predicted and identified.

Cluster analysis of the heatmap provided a comprehensive view of the expression patterns of eleven TF genes among the different samples; the TF genes were differentially expressed and significantly regulated by drought stress and *F. mosseae* inoculation (Figure 8B). Under WW conditions in maize roots, *F. mosseae* inoculation upregulated the expression of *Homeobox127*, *bHLH124*, *NAC22*, *ABI3-VP120*, and HSF28, while downregulated the expression of *bzip40* and *LBD41* transcription factors. In contrast, under WW conditions in maize leaves, *F. mosseae* inoculation induced the expression of *bHLH124*, *MYB115*, *Heat StressC-1*, *NAC22*, *ABI3-VP120* and *HSF28*, and repressed the expression of *bzip40* and *Homeobox127*. Under drought stress conditions in maize roots, *F. mosseae* inoculation upregulated the expression of *bzip40*, *Homeobox127*, *LBD4*, *LBD41*, and *NAC22*, while downregulated the expression of *AP2-EREBP113*, *bHLH124*, *Heat Stress C-1*, *ABI3-VP120*, and *HSF28*. Under DS conditions in maize leaves, *F. mosseae* inoculation induced the expression of *Homeobox127*, *LBD4*, *Heat Stress C-1*, *NAC22*, *ABI3-VP120*, and *HSF28*, and repressed the expression of *bHLH124*.

More interestingly, many Ca^2+^ signaling-related DEGs previously mentioned for their important role in drought stress and *F. mosseae* inoculation responses were found co-expressed together with some TFs. The co-expressed network contained 25 CBL-CIPK genes and 25 TFs (Figure 8C), and another co-expressed network included 19 CDPK-MAPK genes and 23 TFs (Figure 8D). *Whirly1* and *BES1* with CBL-CIPK genes and *B3* with CDPK-MAPK genes were proved to be the highest number of connections within the co-expressed network (Figure 8C,D). Based on the expression data and co-expression analysis, *Whirly1* with *CBL11*, and *BES1* with *CBL10*, *CIPK24*, *CDPK1*, *CDPK14*, *CDPK19*, and *MAPK9* genes showed significant positive correlations, while *B3* with *MAPK1* and both *CBL9* genes showed significant negative correlations in response to both *F. mosseae* inoculation and drought stress.

## 4. Discussion

In our previous studies, maize (cultivar: Zhengdan 958) seedlings were inoculated with *F. mosseae* under WW and DS conditions, and the results indicated that *F. mosseae* successfully established symbiotic relationships with the maize roots. Additionally, *F. mosseae* inoculation significantly enhanced the drought tolerance of maize by promoting growth, improving root hydraulic conductivity, reducing membrane electrolyte leakage and oxidative damage, and notably regulating the expression of aquaporin genes [24]. The above studies revealed the positive effects of *F. mosseae* symbiosis on drought tolerance in Zhengdan 958 maize by using phenomic approaches. In our present study, we focused the investigation on the molecular mechanisms of the Ca^2+^ signaling pathway for mycorrhiza-enhanced drought tolerance in maize roots and leaves by transcriptional analysis. This finding showed that drought stress, *F. mosseae* inoculation, and variations in plant tissues led to a significant alteration in gene expression, yielding a total of 14,865 differentially expressed genes (DEGs) identified through transcriptome sequencing, which were potential target genes for further investigating the response mechanisms and characterizing the related function regulated by drought stress and AMF symbiosis. A total of 189 DEGs obviously responded not only to AMF symbiosis and drought stress, but also exhibited preferential expression in either maize leaves or roots, while the identification of DEGs across the three treatment factors provided novel insights into the role of AMF symbiosis in maize’s response to drought stress. This study represents the first investigation of the differential transcriptomic profile of maize leaves and roots following AMF inoculation under drought stress.

### 4.1. F. mosseae-Induced Ca^2+^ Fluxes and Contents in Maize Roots and Leaves Under Drought Stress

The calcium ions (Ca^2+^), serving as a ubiquitous secondary messenger, transmitted a wide range of environmental stimuli to trigger appropriate physiological responses [25,26,27]. Ca^2+^ was released from both extracellular and intracellular sources into their cytoplasm through Ca^2+^ channels, leading to a rapid increase in cytosolic-free Ca^2+^ concentration ([Ca^2+^]_cyt_) [6]. Changes in [Ca^2+^]_cyt_ were reported in response to various environmental stimuli signals, including hormones, light, abiotic stress, and microbial elicitors, playing a crucial role in the early defense responses of plants [27,28]. Positive values of Ca^2+^ fluxes indicate an outflow, while negative values suggested inflow [6]. Increased Ca^2+^ influx and cytoplasmic Ca^2+^ levels were beneficial for abscisic acid (ABA) transduction in plant guard cells, ultimately enhancing plant drought resistance [29]. In this study, a non-invasive micro-test technique was employed to investigate Ca^2+^ fluxes in maize leaves and roots; *F. mosseae* inoculation significantly decreased the net fluxes of Ca^2+^ in both maize leaves and roots under drought stress, indicating reduced Ca^2+^ outflow and increased Ca^2+^ inflow. Similarly, *F. mosseae* inoculation significantly decreased the net Ca^2+^ fluxes in both *Gossypium* leaves and roots under arsenic stress [30]. In our study, drought stress decreased the Ca^2+^ content in maize roots and leaves, but *F. mosseae* inoculation enhanced Ca^2+^ content in both maize roots and leaves. Wang et al. [31] also observed that *Rhizophagus irregularis* inoculation increased Ca^2+^ content and reduced the Na^+^/Ca^2+^ ratio in *Casuarina glauca* roots under NaCl stress. The enrichment of Ca^2+^ in mycorrhizal plants was associated with the high Ca^2+^ uptake capacity of mycorrhizal fungi hyphae.

### 4.2. AMF-Induced CBL-CIPK Gene Expression in Maize Roots and Leaves Under Drought Stress

B-like proteins (CBL) specifically bound to CBL-interacting protein kinases (CIPKs) to form bimolecular sensor responders in the Ca^2+^ signaling pathway, and they perceived an array of biotic and abiotic stress, including drought, heat, cold, heavy metal, salt stress, and pathogenic fungi; the function of CBL-CIPK genes in abiotic stresses was well-demonstrated diversity [9,32]. The suppression of *GmCIPK2* expression in soybean (*Glycine max*) hairy roots via RNA interference resulted in enhanced sensitivity to drought; conversely, the *GmCIPK2* overexpression in soybean was found to improve drought tolerance in transgenic *Arabidopsis* [33]. The overexpression of *OsCIPK12* could increase the drought tolerance of rice (*Oryza sativa*) at the vegetative stage [34]. Transcriptomic analysis revealed that a total of 8 AcCBL and 21 AcCIPK genes of *Ananas comosus* exhibited differential expression patterns in the diverse tissues and development stages; further, the *AcCBL1* expression in different *A. comosus* tissues were significantly induced in response to an array of abiotic stimuli [35]. Most CBL and CIPK genes showed obvious effects with respect to drought tolerance and AMF inoculation, and the differential expression profile of eight *CsCBLs* found in the root tissues of *Citrus sinensis*, *F. mosseae* inoculation improved the expression of *CsCBL4*, *5*, *6*, and *7*, while repressing the expression of *CsCBL1*, *2*, *3*, and *8* under both WW and DS conditions; additionally, drought significantly downregulated *CsCBL8* and upregulated *CsCBL7* under non-AMF inoculation conditions [9]. The gene expression of *ZmCIPK1*, *3*, *8*, *17*, and *18* in maize leaves and roots was induced by water stress [36]. *ZmCIPK8* was identified as a regulator of maize’s response to drought stress, and overexpression of *ZmCIPK8* in tobacco resulted in enhanced drought tolerance of transgenic tobacco seedlings [37]. In our study, 10 CBLs and 31 CIPK genes were expressed in maize roots and leaves and induced by drought stress and *F. mosseae* inoculation, which indicated that CBL-CIPK genes played a vital role in the related biological processes in response to drought stress and mycorrhizal interactions. Under DS conditions, *F. mosseae* inoculation induced *ZmCBL4*, *5*, *6*, *7*, *10*, *11*, and *12*, and repressed *ZmCBL1*, *2*, and *9* in maize roots, while *F. mosseae* inoculation also induced the expression of *ZmCBL2*, *5*, *6*, *7*, *9*, *10*, *11*, and *12*, and repressed the expression of *ZmCBL1* and *4* in maize leaves, and similar results were shown for ZmCIPKs. These results indicated that the regulation of CsCBL and CsCIPK expression was not consistent with the cis-elements found in their promoters, possibly due to the integration of other gene regulatory components, such as trans-acting factors [9].

### 4.3. AMF-Induced MAPK Gene Expression in Maize Roots and Leaves Under Drought Stress

Mitogen-activated protein kinases (MAPKs), which are members of the serine/threonine protein kinase family, underwent activation in response to extracellular stimuli through the complex MAPK cascade reaction (MAPKKK-MAPKK-MAPK), which regulates various cellular, physiological and biochemical processes, such as plant growth and development, cell division, and hormone response, as well as adaption to a wide range of biotic and abiotic stresses, including drought, salinity, heat and heavy metal stress, and pathogen infection [3,4,37,38]. MAPK cascade genes were documented to exhibit responses to drought stress in various plant species, including *Arabidopsis*, rice, corn, and other plants [39]. Through using RNA interference (RNAi) and virus-induced gene silencing (VIGS) technology, some researchers indicated that MAPK genes exerted a double-sided effect in modulating the drought tolerance of plants: they positively regulated to improve drought tolerance and inversely regulated to increase drought sensitivity [4]. Drought stress rapidly upregulated the expression of *AtMPK3* and *AtMEKK1* in *Arabidopsis* [3], and overexpression of *OsMAPK5* in rice (*Oryza sativa* L.) also had positive responses to drought stress [40]. Under drought stress, *FtMAPK1* and *FtMAPK3* exhibited high expression levels in the roots, stems and leaves of *Fagopyrum tataricum*, *FtMAPK5* showed higher expression levels in the roots, leaves, and flowers, while *FtMAPK8* and *FtMAPK10* were predominantly expressed in the leaves and roots [38]. The expression levels of AMF and soybean MAPKs in mycorrhizal soybean roots were upregulated by drought stress, which indicated that the activation of MAPK signals improved the drought resistance of mycorrhizal soybean [41]. Huang et al. [4] found that *R. irregularis* inoculation significantly upregulated *MdMAPK7-1*, *MdMAPK16-2*, *MdMAPK17*, and *MdMAPK20-1* in *Malus hupehensis* under drought stress. *R. irregularis* inoculation enhanced the drought tolerance of *Populus simonii × P. nigra* by mediating the gene expressions of *PsnMAPK7-2*, *PsnMAPK16-1*, *PsnMAPK19-2*, and *PsnMAPK20-2* in mycorrhizal roots; meanwhile, drought stress induced higher *PsnMAPKs* expression levels in non-mycorrhizal leaves compared to mycorrhizal leaves, suggesting that non-mycorrhizal plants were more sensitive to drought. However, genome-scale transcriptome studies of MAPK genes on mycorrhizal maize under drought stress still need further investigation. In this study, twenty-one differential expression genes of the MAPK family were screened from the maize genome through high-throughput sequencing. *F. mosseae* inoculation induced *ZmMAPK1*, *2*, *3*, *7*, *10*, *11*, and *17*, and repressed the expression of *ZmMAPK3*, *7*, *9,* and *11* in maize roots under DS conditions, while it also induced the expression of *ZmMAPK1*, *2*, *3*, *7*, *9*, *10*, *17*, and *20*, and repressed the expression of *ZmMAPK1*, *9*, *11*, and *18* in maize leaves, which speculated that *F. mosseae* inoculation regulated the MAPK pathways in maize seedlings to alleviate drought stress. Signaling communication between AMF and host plants though the MAPK pathways may be important to the drought tolerance of mycorrhizal maize seedlings. Our findings were significant in understanding the function of MAPK genes of mycorrhizal maizes in response to drought stress.

### 4.4. AMF-Induced CDPK Gene Expression in Maize Roots and Leaves Under Drought Stress

Calcium-dependent protein kinases (CDPKs) frequently mediated Ca^2+^ signaling transduction pathways by either stimulus-dependent activation or directed phosphorylation of functional protein, which play pivotal roles in the plant’s response to abiotic stresses, including drought [10,42]. The CDPK-mediated signaling response to drought stress involves the closure of stomata to reduce water loss by regulating abscisic acid (ABA) signaling and guarding cell ion channels [43]. For example, *Arabidopsis CPK1* upregulation increased the ABA accumulation due to activating NADPH oxidase and phosphorylate phenylalanine ammonia-lyase [44]. *Arabidopsis* CPKs (AtCPKs, CDPKs also termed CPKs) were detected in the guard cells, and the expression of *AtCPK4* and *AtCPK11* was induced by drought stress, which displayed reduced stomatal ABA responsiveness [45]. The *Oryza sativa* CDPKs were induced by drought stress, and *OsCDPK7*, 9, or *13* overexpression increased the rice resistance to drought [46]. CDPK expression also related to the response to microbe interactions, and *OsCPK10* expression was induced by a *Magnaporthe grisea* elicitor, but *OsCPK12* oppositely modulated salt-stress tolerance and blast disease [47]. Upregulation of *OsCPK9* expression enhanced the drought tolerance of rice by improving stomatal movement and osmotic adjustment, thus reducing water loss; additionally, both overexpression and RNA interference of *OsCPK9* regulated the transcript levels of ABA- and stress-responsive genes. [41]. Shu et al. [9] found that twenty-nine *Citrus sinensis* CDPK were also expressed in citrus roots, seventeen of them were upregulated, and *CsCDPK3*, *7*, and *28* were downregulated by *F. mosseae* inoculation; furthermore, *CsCDPK20* and *CsCDPK22* were downregulated by *F. mosseae* inoculation under WW conditions, but upregulated by *F. mosseae* inoculation under DS conditions. However, the literature on different CDPK expression in plant root and leaf response to AMF colonization and drought stress was limited. In our study, *F. mosseae* inoculation, tissue difference, and drought stress exerted respective or synergistic effects on the gene expression of maize CDPKs, and the eleven ZmCDPK genes in maize seedlings were found to express and exhibit the distinct expression patterns due to the above three factors. Under *F. mosseae* inoculation conditions, drought stress induced the expression of *ZmCDPK1*, *2*, *3*, *8*, *11*, *13*, and *14*, but repressed *ZmCDPK4*, *5*, *7*, and *19* in maize roots, *ZmCDPK2* was upregulated, but *ZmCDPK1*, *3*, *4*, *5*, *7*, *8*, *11*, *13*, *14*, and *19* were downregulated in maize leaves, which indicated that multiple CDPKs expression in plants roots and leaves played an important role in intricate Ca^2+^ signal transduction during drought and AMF–plant interactions [9].

### 4.5. Regulation of Transcription Factors by F. mosseae Inoculation Under Drought Stress

Transcription factors (TFs) represent a class of protein sequences, and activate or repress the transcription of nearby genes; they regulate the transcription rate of genetic information from DNA to mRNA, which is related to cellular processes, development, differentiation, and response to stimuli in plants [48]. Many specific TFs have a crucial role in regulating mycorrhiza-induced response in host plants to environmental stress, as they are subject to distinct transcriptional regulation and possess the capability to amplify cellular signals in response to specific stimuli [49]. TFs, specifically those belonging to the GRAS, AP2/ERF, and MYB family, are upregulated by AMF symbiosis, thereby participating in stress regulation through hormones and other molecular signaling pathways [17]. In this study, eleven TFs showed differential expression patterns in different tissues of maize seedlings due to *F. mosseae* inoculation and drought stress. *F. mosseae* inoculation upregulated the expression of Homeobox 127, LBD4, and NAC22, while it downregulated bHLH124 expression in both maize roots and leaves under drought stress, suggesting that the coordinated regulation of TFs was a mechanism employed by maize leaves and roots in response to AMF colonization under drought stress conditions. NAC overexpression in *Oryza sativa* L., *Arabidopsis*, and *Nicotiana benthamiana,* respectively, was proven to improve abiotic and biotic stress responses of plants [50,51,52,53]. Wang et al. [54] also demonstrated that the expression of NAC enhanced the defense responses of tomato seedlings against necrotrophic fungal and bacterial pathogens. AMF inoculation was found to regulate seventeen wheat TFs, which included an NAC68, and two bHLH. *F. mosseae* inoculation enhanced the As tolerance of *Gossypium* seedlings through the regulation of 34 TF families, notably including bHLH, ERF, MYB, MYB-related, NAC, and WRKY families, and among these, a significant number of NAC TFs were associated with cell wall development and the extent of lignification [30]. In this study, *F. mosseae* inoculation induced the *MYB115* expression under well-watered conditions in maize leaves. Tao et al. [30] also found that *PsnMAPK7-1*, *PsnMAPK19-1*, and *PsnMAPK20-2* genes induced by AMF inoculation acted through MYB transcription factors to enhance drought tolerance of mycorrhizal *Populus simonii* × *P. nigra*. In our study, specific TFs were found to co-express together with previously mentioned DEGs; for instance, *BES1* was co-expressed with *CBL10* and *CBL24*, and *B3* was connected to *CBL2*, *CBL5*, *CBL9*, *CBL10*, and *CIPK24*. These TFs may be related to the drought tolerance mechanism of mycorrhizal maizes through Ca^2+^ signaling pathway networks.

## 5. Conclusions

In our previous studies, *F. mosseae* established symbiotic relationships with maize roots under well-watered (WW) and drought-stressed (DS) conditions, and reports also indicated the positive effects of *F. mosseae* inoculation on maize growth, root hydraulic conductivity, reduction in membrane electrolyte leakage, mitigation of oxidative damage, and the expression of aquaporin genes in maize [25]. In this study, *F. mosseae* inoculation increased the net Ca^2+^ fluxes and Ca^2+^ contents in the roots and leaves of *Gossypium* under both WW and DS conditions. In order to elucidate the molecular mechanisms of the Ca^2+^ signaling pathway underlying the drought tolerance of maize regulated by AMF, the differentially expressed maize genes of the Ca^2+^ signaling pathway induced by *F. mosseae* inoculation under drought stress were comprehensively investigated by using high-throughput transcriptomics techniques. The results showed that 189 differentially expressed genes (DEGs) were regulated not only by AMF symbiosis and drought stress, but also exhibited preferential expression in either leaves or roots. The annotation and enrichment of Gene Ontology (GO) and the Kyoto Encyclopedia of Genes and Genomes (KEGG) showed that most of these DEGs were significantly enriched in Ca^2+^ signaling pathway genes, related to signal transduction, cellular process, and defense response. A high number of DEGs with this function (including CBL, CIPK, MAPK, and CDPK receptor kinases) were up-DEGs or down-DEGs in *F. mosseae*-inoculated maizes under drought stress. Furthermore, many transcription factor families, mainly including ERF, MYB, and bHLH, were speculated to play key roles in improving the drought tolerance of maize. Based on the expression data and co-expression analysis between transcription factor and Ca^2+^ signaling pathway genes, *Whirly1* with *CBL11*, and *BES1* with *CBL10*, *CIPK24*, *CDPK1*, *CDPK14*, *CDPK19*, and *MAPK9* genes showed significant positive correlations, while *B3* with *MAPK1* and both *CBL9* genes showed significant negative correlations in response to both *F. mosseae* inoculation and drought stress. The regulation of Ca^2+^ signaling pathways by AMF symbiosis was an important response mechanism of maize to improve their drought resistance. This study provided valuable insights into the modulation of gene expression in the Ca^2+^ signaling pathway induced by AMF, which has the potential to enhance drought tolerance of mycorrhizal maize in the future.

## Figures and Tables

**Table 1:** Analysis of transcriptome sequencing and gene annotation.

**(A) Statistics of Transcriptome Sequencing**										
**Sample**	**Raw Reads**	**Clean Reads**	**Clean Reads** **Ratio (%)**	**Q20%**	**Q30%**	**GC%**	**Total Reads**	**Total** **Mapped**	**Multiple Mapped**	**Uniquely** **Mapped**
AWR_3	49004258	48267150	0.98	97.74	93.78	52.69	48267150	39998058(82.87%)	2246287(4.65%)	37751771(78.21%)
AWR_2	43278510	42415960	0.98	97.88	94.07	52.59	42415960	28480510(67.15%)	1675997(3.95%)	26804513(63.19%)
AWR_1	44510978	43872336	0.99	97.83	93.98	52.26	43872336	34821451(79.37%)	3450861(7.87%)	31370590(71.5%)
AWL_3	49623792	48947880	0.99	97.74	93.8	53.91	48947880	42329513(86.48%)	2918391(5.96%)	39411122(80.52%)
AWL_2	41304944	40581566	0.98	97.69	93.84	53.36	40581566	32763271(80.73%)	2481943(6.12%)	30281328(74.62%)
AWL_1	55064656	54262806	0.99	98.16	94.67	51.53	54262806	42882808(79.03%)	1924899(3.55%)	40957909(75.48%)
ADR_3	46985536	46484262	0.99	97.98	94.3	53.55	46484262	37870479(81.47%)	1800566(3.87%)	36069913(77.6%)
ADR_2	43801530	43222778	0.99	98	94.3	52.01	43222778	35468866(82.06%)	1715287(3.97%)	33753579(78.09%)
ADR_1	46029238	45542410	0.99	97.93	94.13	53.15	45542410	39682609(87.13%)	1576485(3.46%)	38106124(83.67%)
ADL_3	46078662	45479426	0.99	98.03	94.43	53.75	45479426	38905176(85.54%)	3218143(7.08%)	35687033(78.47%)
ADL_2	41448764	40812670	0.98	97.97	94.3	52.4	40812670	32832809(80.45%)	2032086(4.98%)	30800723(75.47%)
ADL_1	44821098	44248464	0.99	97.82	93.91	53.71	44248464	37521200(84.8%)	2519531(5.69%)	35001669(79.1%)
CKWR_3	41299570	40841682	0.99	97.97	94.23	51.98	40841682	33667906(82.44%)	1656725(4.06%)	32011181(78.38%)
CKWR_2	44071930	43511138	0.99	97.8	93.87	51.96	43511138	34440119(79.15%)	1427552(3.28%)	33012567(75.87%)
CKWR_1	44310396	43754414	0.99	97.89	94.01	51.74	43754414	34045241(77.81%)	1375476(3.14%)	32669765(74.67%)
CKWL_3	45821846	45246014	0.99	97.92	94.16	53.51	45246014	36643359(80.99%)	3637806(8.04%)	33005553(72.95%)
CKWL_2	47213008	46625466	0.99	98.08	94.53	53.53	46625466	39198700(84.07%)	2417415(5.18%)	36781285(78.89%)
CKWL_1	43116158	42534804	0.99	98.02	94.41	53.24	42534804	34386366(80.84%)	3106046(7.3%)	31280320(73.54%)
CKDR_3	46665196	46112606	0.99	97.94	94.17	53.12	46112606	33263877(72.14%)	1249731(2.71%)	32014146(69.43%)
CKDR_2	45748734	45274614	0.99	98.07	94.52	53.74	45274614	38031581(84.0%)	2062495(4.56%)	35969086(79.45%)
CKDR_1	41104330	40618086	0.99	97.99	94.29	52.39	40618086	34492466(84.92%)	1618976(3.99%)	32873490(80.93%)
CKDL_3	40917860	40519570	0.99	98.13	94.63	53.74	40519570	34016680(83.95%)	2245287(5.54%)	31771393(78.41%)
CKDL_2	43367096	42950066	0.99	98.11	94.61	53.62	42950066	34668701(80.72%)	2233725(5.2%)	32434976(75.52%)
CKDL_1	44047556	43590294	0.99	98.05	94.49	54.14	43590294	34692789(79.59%)	2555542(5.86%)	32137247(73.73%)
**(B) Annotation Analysis of Expressed Genes and Transcripts**										
**DB Name**		**Expressed Gene Number** (**Percent**)		**Expressed Transcript Number** (**Percent**)			**All Gene Number** (**Percent**)		**All Transcript Number** (**Percent**)	
GO		35283(0.7551)		138412(0.8598)			40189(0.7059)		154937(0.8424)	
KEGG		16514(0.3534)		80595(0.5006)			18538(0.3256)		89473(0.4865)	
COG		37828(0.8096)		149246(0.9271)			42635(0.7489)		166372(0.9046)	
NR		42327(0.9059)		155685(0.9671)			48510(0.8521)		174495(0.9488)	
Swiss-Prot		28570(0.6114)		125447(0.7792)			31367(0.5509)		138779(0.7546)	
Pfam		29227(0.6255)		116275(0.7223)			31933(0.5609)		127784(0.6948)	
Total annotation		42446(0.9084)		155829(0.968)			48662(0.8547)		174675(0.9497)	
Total		46725(1.0)		160987(1.0)			56933(1)		183919(1)	

## Data Availability

The original contributions presented in the study are included in the article, further inquiries can be directed to the corresponding author.
